# Dataset size versus homogeneity: A machine learning study on pooling intervention data in e-mental health dropout predictions

**DOI:** 10.1177/20552076241248920

**Published:** 2024-05-15

**Authors:** Kirsten Zantvoort, Nils Hentati Isacsson, Burkhardt Funk, Viktor Kaldo

**Affiliations:** 1 Institute of Information Systems, 27720 Leuphana University, Lueneburg, Germany; 2 Centre for Psychiatry Research, Department of Clinical Neuroscience, 27106 Karolinska Institutet, & Stockholm Health Care Services, Stockholm, Sweden; 3 Department of Psychology, Faculty of Health and Life Sciences, 4180 Linnaeus University, Växjö, Sweden

**Keywords:** e-mental health, dropout, machine learning, prediction, ICBT

## Abstract

**Objective:**

This study proposes a way of increasing dataset sizes for machine learning tasks in Internet-based Cognitive Behavioral Therapy through pooling interventions. To this end, it (1) examines similarities in user behavior and symptom data among online interventions for patients with depression, social anxiety, and panic disorder and (2) explores whether these similarities suffice to allow for pooling the data together, resulting in more training data when prediction intervention dropout.

**Methods:**

A total of 6418 routine care patients from the Internet Psychiatry in Stockholm are analyzed using (1) clustering and (2) dropout prediction models. For the latter, prediction models trained on each individual intervention's data are compared to those trained on all three interventions pooled into one dataset. To investigate if results vary with dataset size, the prediction is repeated using small and medium dataset sizes.

**Results:**

The clustering analysis identified three distinct groups that are almost equally spread across interventions and are instead characterized by different activity levels. In eight out of nine settings investigated, pooling the data improves prediction results compared to models trained on a single intervention dataset. It is further confirmed that models trained on small datasets are more likely to overestimate prediction results.

**Conclusion:**

The study reveals similar patterns of patients with depression, social anxiety, and panic disorder regarding online activity and intervention dropout. As such, this work offers pooling different interventions’ data as a possible approach to counter the problem of small dataset sizes in psychological research.

## Introduction

Modern societies struggle to provide adequate mental health care,^1–3^ as traditional therapy alone is not meeting the increasing need.^4^ Internet-based Cognitive Behavioral Therapy (ICBT) promises to improve care levels by achieving similar goals as face-to-face therapies through efficient digital means.^2,5^ With the rise of ICBTs, a large variety of user online activity data becomes recordable. Applying advanced analytics to this data holds great promise to individualize and improve care.^6–8^ One task that presents itself to be solved with Machine Learning (ML) is that of intervention dropout predictions.^4,6^ A patient dropping out of an intervention is significantly less likely to have positive outcomes.^9^ Yet, upon starting the intervention, costs occur, and scarce resources are occupied.^10^ Measures such as guidance from therapists lower dropout rates,^11^ but are often too costly to be provided to all patients.^12^ Identifying patients at risk of dropout early on allows for the personalized allocation of measures. If more individuals’ needs for support are met, resource allocation is optimized, and health benefits increase.^13^ In contrast to the direct prediction of health outcomes, dropout predictions include patients that otherwise tend to be ignored due to missing symptom data.^14,15^

Initial studies using ML to predict dropout based on user behavior data show promise.^6,16–21^ Nevertheless, there are still few ML applications in ICBTs, especially dropout predictions.^16,21–24^ A recent systematic literature review found that, despite the widespread consensus about its value, only 3 out of 94 digital interventions use ML to personalize interventions for depression.^8^ This scarcity of work is attributed to the small size of available datasets for training,^8,25^ as it limits the accuracy and generalizability of predictions.^26–28^ Collecting large health datasets is costly,^29^ and the median dataset size across 59 studies using ML for outcome predictions in depression treatments was found to be only 115 patients.^30^ Similarly, the median for related work predicting dropout in online interventions was 342 patients.^6,16–21^ Thus, with ML approaches in ICBTs, the question arises of how to produce accurate predictions despite small datasets.^7,23,31^

Albeit the lack of large datasets, the number of smaller datasets available was already reported to be in the hundreds 5 years ago.^32^ Data sharing between providers creates larger training datasets but poses significant challenges regarding data privacy, data interoperability, and conflicting interests.^33^ However, many providers themselves offer similar interventions for different but related primary symptoms, such as depression and anxiety disorders.^20^ As symptoms and behavior are interconnected,^34^ common behavioral patterns between patients could be leveraged by pooling several interventions into one dataset. If successful, this not only improves prediction results but also lowers model maintenance efforts. However, pooling the data may be detrimental if contexts and user behaviors differ significantly. Most papers predicting dropout focus on a single intervention,^6,16,17,20,21^ while one gathers two different interventions for the same symptoms,^19^ and one gathers interventions for three different symptoms.^18^ While this shows that different options are possible, no study comparatively evaluates the approaches. It, generally, remains unclear how patients with different but related target symptoms vary in their online intervention behavior. Clustering analyses have successfully identified user archetypes in mental health apps in general^35^ and within specific interventions.^36^ Applying such a clustering analysis to users of different but related ICBTs could offer valuable insights into the similarities in user behavior.

In this study, we use demographic, symptom, and online user behavior data of 6418 routine care patients from the Internet Psychiatry in Stockholm, Sweden. The main research question is if the value of pooling intervention data for social anxiety (SAD), major depression (MDD), and panic disorder (PD) outweighs the downside of losing data homogeneity when predicting intervention dropout. The goal is to identify patients who will end up leaving the intervention early without benefiting, already in intervention week 4 of 12. For this, four different supervised ML methods (i.e. Logistic Regression, Support Vector Machines, Random Forest, and AdaBoost classifiers) are compared. We further investigate the relationship between prediction performance and dataset size. To this end, the training on individual versus pooled datasets is repeated on samples of the median dataset sizes of related work. A clustering analysis is the intermediate step to understanding the differences and similarities between the interventions’ data. Through these proposed steps, this study aims at (1) exploring the heterogeneity of online intervention data across three highly prevalent mental disorders and (2) providing insights into what pooling these different intervention datasets into one dataset yields for dropout prediction. Finally, this work adds to the limited body of research investigating dropout predictions across three large routine care interventions.

## Methods

### Interventions and participants

This study uses routine care outpatient data from the Internet Psychiatric clinic in Stockholm, Sweden from 2008 to 2020.^37^ The data comprises all available patients undergoing treatment while the platform in question was used. The datasets consist of 1633 PD, 1907 SAD, and 2902 MDD patients. The treatments have previously been evaluated with positive results.^38–40^ After a psychiatric assessment, each patient received 12 weeks of disorder-specific intervention. The assessment and treatment evaluation are based on established patient self-rating measures; for PD, the Panic Disorder Severity Scale-Self Report^41^; for SAD, the Liebowitz Social Anxiety Scale-Self Report,^42^ and for MDD, the Montgomery–Åsberg Depression Rating Scale-Self Report^43,44^ were used. Each of the three interventions was administered in the same clinical context where patients self-refer and then go through a web-based screening and the established semistructured M.I.N.I diagnostic assessment interview^45^ with a psychiatrist. During the interview, the clinician provides information about (I)CBTs in general, and the expected effort required from the patient to complete treatment. As such, all patients are ensured to have a relevant diagnosis as well as being informed about the treatment, and sufficiently motivated to engage in it.

Included patients receive the same therapist support routine across all three interventions. The interventions reside on the same technical platform and are very similar in structure, but clearly differ in therapeutic content. All interventions start with psychoeducation and consist of cognitive behavioral therapy techniques specific for each condition divided into 10 modules. For example, the SAD and PD interventions include symptom-specific exposure exercises, whereas the MDD has a focus on behavioral activation. Each intervention's modules consist mainly of exercises, including homework, messages from and to a therapist and weekly symptom assessments. More detailed information about the interventions is summarized in Supplemental Appendix 1 and has previously been published for SAD,^38^ depression,^39^ and PD.^40^

### Features

The prediction is based on the first 4 weeks of data as a trade-off between gathering sufficient data versus maintaining sufficient time to intervene to prevent dropout.^6^ A previous study has shown personalizing the support level in week 4 to have a positive effect for patients at risk.^13^ For the features, thus, all data gathered after week 4 is disregarded to prevent target leak. First, we include the common sociodemographic variables, age, and gender.^21^ Second, given their importance,^8,44^ the symptom measures at screening, and at the beginning of weeks 1, 2, 3, and 4, respectively, are included. In addition to the actual scores, the time needed to fill out the questionnaires is included. Third, we use the basic information of the intervention set-up (i.e. year of start, start in winter or summer, and target disorder).^21^ Fourth, the character length of the homework assignments and messages are each summed per week, which have previously been found to be predictive of both dropout and health outcomes.^46^ To account for the therapist messages, the percentage of characters sent in the conversation produced by the therapist is included. Following the insights from Bremer et al.,^6^ several features are generated from patients’ log data. This includes the sum of time spent on the intervention, the number of pages clicked, the number of sessions, and number of days that patient logged in. In addition, the time patterns of the login behavior are gathered across all weeks by looking into the percentage of sessions per weekdays and daytimes. Further, we record how many days a patient needed to finish each module. Further information on the preprocessing and feature engineering steps can be found in Supplemental Appendix 2.

The operationalization of the dropout variable is aimed at identifying the patients who are most likely to leave the intervention too early to sufficiently benefit.^47^ The intended symptom improvement is determined by either the final symptom score below the absolute cutoff for remittance (8 for PDSS-SR,^48^ 35 for LSAS-SR,^49^ and 11 for MADRS-S^50^) or a 50% improvement since the start of the treatment.^51^ If no symptom score after week 8 is available, their symptom improvement is classified as “unknown.” Module completion has been found to be the adherence measure with most consistently positive power toward explaining therapy outcome.^52^ For this study, we use module 8 of 10 as it contains all unknown leavers, it includes all of the content introductions, as the last 2 modules are repetition and maintenance,^52,53^ and produces considerably balanced classes. A more detailed explanation of the dropout variable can also be found in Supplemental Appendix 2. The averages, SD and units of the resulting dataset can be found in Supplemental Appendix 3. All steps, including the subsequent modeling, are implemented in Python, using the pandas,^54^ Numpy,^55^ Kneed algorithm,^56^ and Scikit-learn^57^ libraries.

### Exploration of heterogeneity between interventions

This analysis addresses the first goal: the exploration of heterogeneity in patients across the three interventions using the demographic, intervention, symptom, online activity, and character counts variables. The general purposes of clustering are to gain insight into the data, identify natural groups, and be able to summarize them based on segment prototypes.^58^ First proposed in 1967, k-means algorithms are among the most used clustering approaches due to their computational efficiency and easy implementation.^58,59^ The k-means algorithm is an optimization algorithm that iteratively finds a set of k centroids, such that the total sum of distance between each point and its nearest centroid is minimized.^60^ As such, it optimizes for groups that are as similar as possible within themselves but as different as possible from each other.^60^ The number of clusters needs to be decided apriori and is inferred using the Elbow (or Knee) method.^61^ In essence, this method uses the explained data variance to reveal where the marginal gain of a new cluster is outbalanced by the increasing number of clusters. As commonly done, a Principal Component Analysis is conducted prior to the clustering to lower the number of dimensions, reduce multicollinearity and facilitate the visualization of clusters.^62^ The number of principal components is also automatically identified by using the Kneed algorithm.^56^ The critical question is whether the algorithm will rely on the target disorder variable identifying each intervention as primary splitting criteria. If the different target symptoms result in different online behavior, the clustering algorithm would be expected to reproduce the three intervention groups. Only if patients behave sufficiently similar across interventions, mixed clusters can be expected. To ensure comparability, this process is done only on the first 4 weeks of data also available to the prediction models. This excludes the dropout and health outcome variable for all patients, which will, however, be added after completing the clustering for the evaluation of clusters.

### Dropout prediction

The second goal is investigating the effects of pooling patients from interventions for depression, SAD, and PD to one dataset when predicting dropout. To this end, models trained on each of the interventions’ data individually are compared to models trained on all three interventions pooled. As the data has almost twice as many MDD as PD patient, we add a pooled run where we under sample the large interventions to have balanced ratios of one-third each. The training dataset sizes at hand are in the four digits, which is already unusually big.^30^ To increase the usefulness of results for future studies, the training process is repeated on smaller samples—the median dataset sizes of related work for outcome prediction (115)^30^ and dropout prediction (342).^6,16–21^ Taking away an assumed 15% data for a holdout test set results in training data of 98 and 291 patients per single intervention.

While the training data differs in dataset size, all models are evaluated on the same 20% stratified test set to maintain comparability across runs. The final evaluation is done (1) relatively and (2) absolutely, both focusing on balanced accuracy (BACC). For the relative evaluation, the single versus pooled data results are compared. For the absolute comparison, the benchmark of (1) better than chance and (2) 67% balanced accuracy as minimally necessary to be valuable in an ICBT to adapt treatment as proposed by Forsell et al.^12^ are used. Further, to adhere to the standards of medical ML studies, accuracy, balanced accuracy, specificity, recall, and area under the curve (AUC) are provided for the test set performances.^63^

In terms of algorithms, Logistic Regression (LR), Support Vector Machines (SVMs),^64^ Random Forest (RF), and AdaBoost^65^ classifiers are chosen as they cover a range from simple and robust to more sophisticated and flexible options.^31,62^ As extensively argued,^66,67^ choosing the algorithm to use in the same step as optimizing the hyperparameters comes with a significant risk of overfitting. Therefore, the model selection will be done through 5 × 10 nested-cross-validation (CV). The inner CV ptimizes hyperparameters via grid search, and the outer CV score determines the one algorithm to use. That algorithm is then retrained on the whole training data with a 10-fold CV, returning a single model to be evaluated on the test set. An intervention-based scaler is added to the pipeline, such that a standard scaler is fitted to the training data per intervention and then applied on the respective holdout fold.^63^

To choose the range of hyperparameters, initial values are run and added to if the outer points seem too low or high. For the algorithms that allow for balancing class weights, the class weights are balanced. For the LR, the choice of L1 and L2 feature selection is optimized as a hyperparameter for the liblinear solver.^68^ The C value is searched across the range [0.001, 0.01, 0.05, 0.1, 0.20, 1]. The SVMs optimize over an RBF and a linear kernel with respective C values [0.001, 0.01, 0.1, 0.25, 0.5, 1]. The RF model searches across the number of estimators [5, 10, 25, 50, 500, 1200], the minimum samples [10, 25, 50, 100, 200], the maximum depths [5, 25, 50, 100, 500, 750], and a binary indicator for bootstrapping. Lastly, the AdaBoost Classifier trades off the number of estimators [1, 2, 5, 10, 25, 100, 1500] with their respective learning rate [0.001, 0.01, 0.1, 1, 2, 2.5].

## Results

### Data heterogeneity

**Figure 1.**
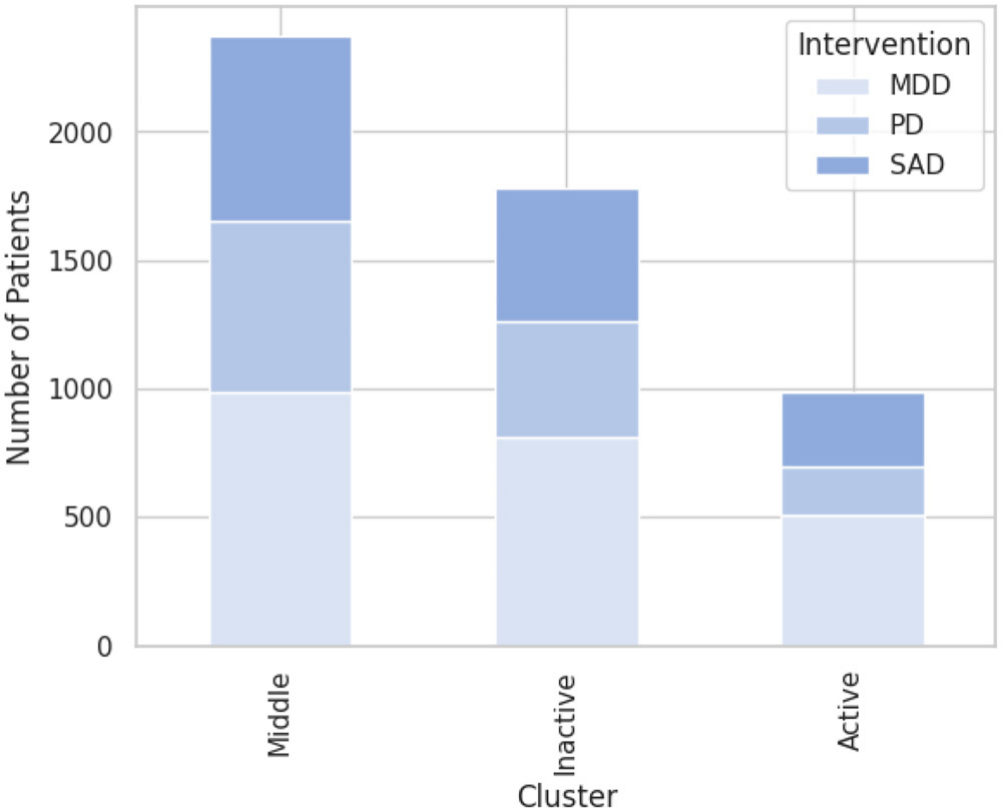
Distribution of patients per intervention and cluster. MDD: major depressive disorder; PD: panic disorder; SAD: social anxiety disorder.

To better understand the differences in online behaviors, user characteristics and symptom patterns, the 1631 PD, 1906 SAD, and 2881 MDD patients and their 57 input variables that result from preprocessing as described in Supplemental Appendix 2 are clustered. The kneed method suggests four principal components to represent the input data. Feeding these components into the k-means algorithm with k ranging from one to 11 suggests 3 most prevalent clusters. However, this optimal value only coincides with the number of interventions, as each intervention spreads comparatively evenly across clusters (Figure 1). The biggest intervention group, MMD, makes up 42–51% of each cluster, SAD accounts for 29–30%, and the smallest intervention, PD, spreads at 20–28% per cluster. To facilitate understanding, the clusters will be referred to as active, middle, and inactive clusters from now on for the reasons explained below. The middle cluster is by far the biggest as it contains 46% of all patients, with the inactive cluster following at 35% and the very active cluster tailing at 19%. All cluster means reported in this section can also be found in the overview table in Supplemental Appendix 3.

Inactive patients are more than 6 times as likely to have missing symptom scores (0.84/5) in the first 4 weeks as active patients (0.13), who are similar to the middle cluster (0.16). Further, the average lengths of messages and homework in the first weeks are 6 times as high for the active cluster (459/1331 characters) as for the inactive cluster (81/224 characters), with the middle clusters averaging at 157/1008 characters. Similarly, login data such as sessions, pages and duration per week are almost all 2–4 times as high for active as inactive patients, with the middle cluster somewhere in-between. The most extreme differences are in the durations where inactive patients, on average, spent one-third or one-fourth of the time (12,071, 6698, 6198, and 6878 s per weeks 1–4) that active patients (32,393, 25,850, 24,738, and 24,171) spent. This is also reflected in the average number of modules completed in the first 4 weeks, with 2.7 for inactive, 4.2 for the middle, and 4.7 for the active patients.

While the starting symptom scores barely differ, inactive patients have a higher average symptom improvement between screening and treatment start (−12%) than middle (−9%) or active (−8%) patients. However, this changes in the following weeks. While inactive patients still see 12% improvement in week 2, they have next to no change (0%, −1%) in week 3 or 4. While the strength of change also lessens for middle and active patients, they still continuously improve (middle: −16%, −3%, −4% and active: −15%, −4%, −5%). The least differentiating variables are the start year, time variables (e.g. time and weekday of intervention use), symptom questionnaire duration, if they started the intervention in the winter, and the patient's age and sex. Retrospectively joining the dropout variable to the clustered data shows that patients from the active cluster are less than half as likely to drop out (25%) as patients from the inactive cluster (65%), with the middle cluster being closer to the active cluster (36%). Despite the dropout rates heavily differing across interventions (PD 28%, MDD 45%, and SAD 57%), the differences in dropout probability per cluster remain. Dropout ratios for the inactive, middle, and active groups are 68%, 36%, and 24% for MDD, 41%, 23%, and 16% for PD, and 80%, 49%, and 33% for SAD. Doing the same for the health outcomes shows that 53% of active and middle patients are treatment successes while only 34% of inactive patients are. For 15% of inactive patients, their health outcome is unknown, whereas the middle cluster has 5% and the active cluster only 2%. As a result, the percentage of not successful treatments is close together between active (45%), middle (43%), and inactive (50%) patients.

### Prediction

**Figure 2.**
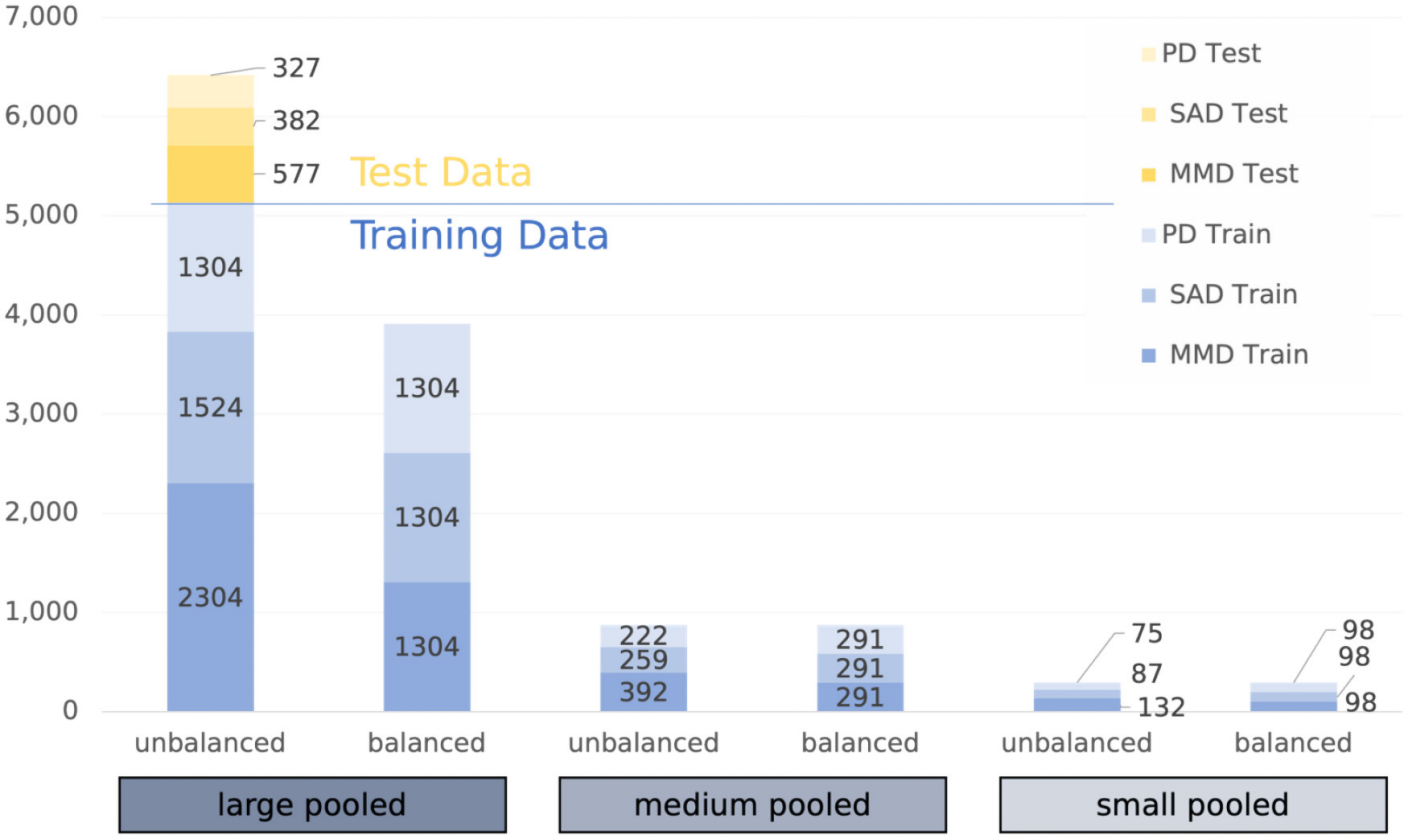
Training and test data sample per pooled run. MDD: major depressive disorder; PD: panic disorder; SAD: social anxiety disorder.

The train-test split leads to a maximum of 5132 training data points and 1289 test data points, for which the averages of all variables can be found in Supplemental Appendix 3. Of these data points, 45% are MDD, 30% are SAD, and 25% are PD patients, resulting in the unbalanced pooled training datasets in Figure 2. The small and medium balanced pooled training data have the same total as the unbalanced run; however, they have balanced ratios of one-third per intervention. For the large data, balancing is dictated by the smallest intervention (PD), resulting in a sample size of 1304 each. The nine single intervention runs (three per disorder) with 98, 291, and 1304/1524/2303 are not separately shown in Figure 2.

**Figure 3.**
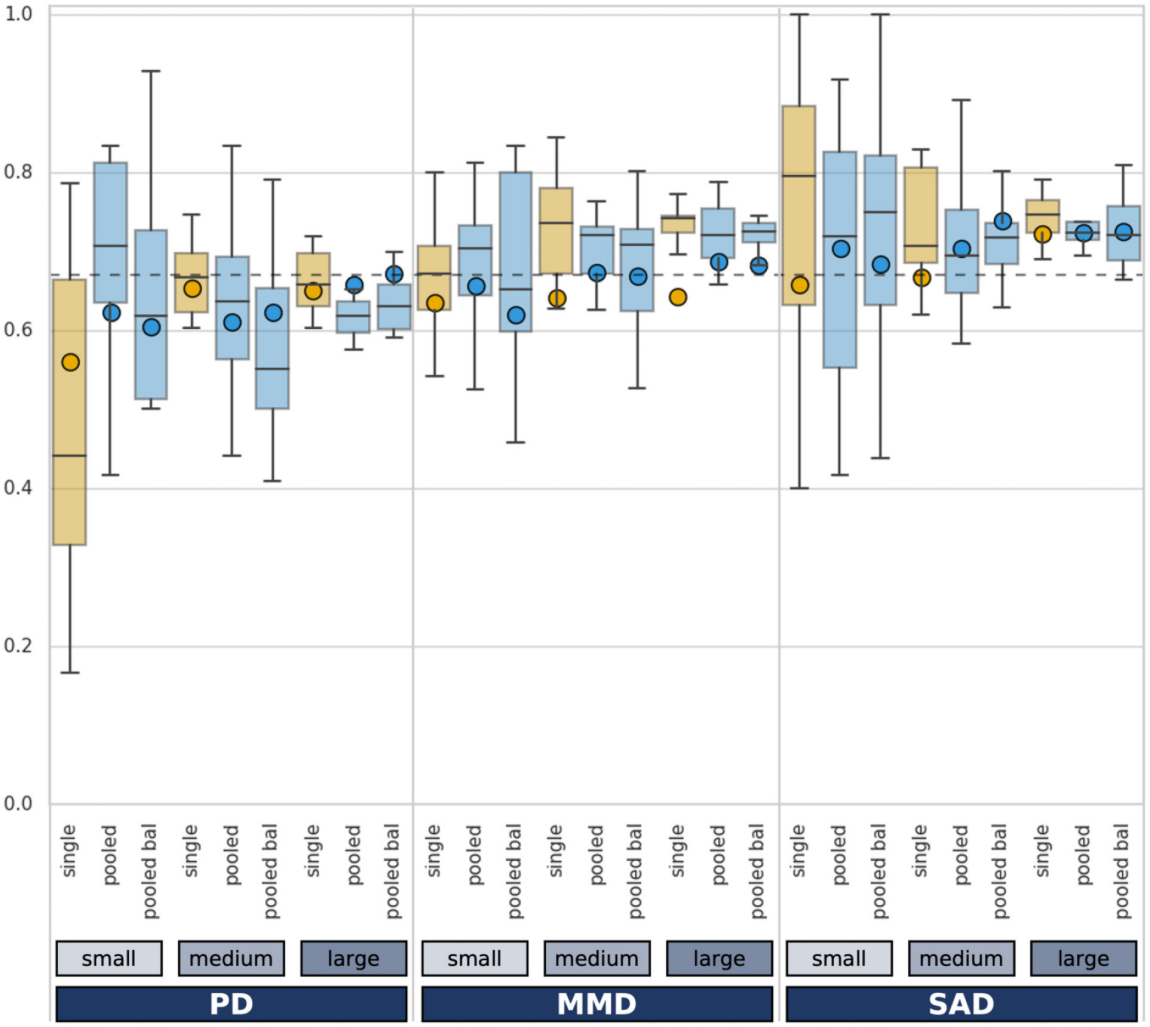
Balanced accuracy for training and test results per run with clinical threshold as dotted line. Bal: balanced intervention shares; MDD: major depressive disorder; PD: panic disorder; SAD: social anxiety disorder.

Figure 3 presents the BACC results for each intervention, dataset size, and type of training data. The box plots show the 10 outer CV scores of the training data, while the single bullet point shows the performance on the test set. An ideal result graph has a high y-axis value (balanced accuracy) with a narrow boxplot (low variance in training results). The boxplot's horizontal stripe (median) should be close to the test set point (neither overfitting nor unexpectedly high results). For pooled data results in the following, the unbalanced (UB) results are mentioned first, followed by the balanced (B) results. To answer the main research question, the single dataset runs are compared to their respective pooled counterparts (e.g. 98 data points single intervention vs 3 × 98 = 294 data points pooled run). All results, including the numbers discussed here, accuracy, recall, specificity, AUC score, and the model type chosen in the CV can be found in the result table in Supplemental Appendix 4.

#### Training data results

Only looking at training results, single runs outperform the pooled datasets in predicting dropout. The single intervention runs have a higher median outer CV score than both pooled runs in six out of nine cases with an average advantage of 0.037 BACC. Further, single runs are higher than at least one pooled run in two more cases. Hence, the only exception is the small (98 data points) PD dataset, where training results for the single run are lower than both pooled data BACCs. For MDD, the median CV score increases as the dataset size increases from small to medium to large, with a total difference of +0.04 BACC. The opposite pattern is visible for PD, such that the training scores decrease as more data is added with −0.05 BACC. Similarly, SAD has the highest score for the small data but then has the smallest score for the medium and an average score for the large data with a total range of −0.08. Considering the range between the first (Q1) and third (Q3) quartile of the CV scores, pooled runs have a lower average range than single interventions. The spread of the training results (Q3–Q1) is lowest for the large datasets at an average of 0.045 in BACC. For the small datasets, it is more than four times as much (average at 0.202), with the medium dataset size in-between (average 0.101).

#### Test data results

Which setting (single vs pooled) performs best is reversed when looking at the test instead of training results. Here, in seven out of nine cases, both pooled runs outperform the single intervention. Additionally, the unbalanced pooled data outperforms the single intervention for the smallest MDD run (0.64 vs UB 0.66/B 0.62). Hence, the single intervention is only superior in one case, the medium PD run. As such, PD patients have both one of the biggest gains and biggest loss (+0.063/−0.044 BACC) when using the pooled model instead of one trained on the PD patients alone. The runs with all available PD data barely differ (+0.007 for pooled). For SAD patients, the small data gains somewhat from pooling (UB +0.046/B +0.026), the medium dataset size has an average and the highest gain (+0.037/+0.072), and there is barely any difference in the large data (+0.000/0.002). On the contrary, MDD patients almost always benefit from pooling the data, and the gains grow with the dataset size (small data UB +0.021/B −0.016, medium data +0.032/+0.028, large data +0.045/+0.040). Keeping the natural ratio is superior for all three small data runs and the medium and large data MDD runs. Balancing the data is favorable for all medium and large datasets of SAD and PD. The largest impacts of balancing the data are −0.037 BACC for the small MDD and +0.035 for the medium SAD datasets. For MDD and SAD, the pooled data's higher BACC also means a higher recall than the single intervention datasets. However, in the case of PD, the pooled datasets have high specificity (average 0.80) but lower recall (average 0.46) whereas the single intervention runs are more balanced (average specificity: 0.57, recall: 0.67).

#### Difference between training and test results

In terms of potential overfitting, the pooled runs have a smaller absolute difference between the test and training results in seven out of nine cases. The only exceptions are the medium and large PD datasets. This results in an average absolute train-test difference in BACC of 0.063 for single and much lower 0.034/0.037 (UB/B) for pooled data. The smallest dataset of SAD has the largest gap (−0.14) with the highest training results out of all runs but the lowest test results of all SAD models. Similarly, the training and test results of the MDD models are twice as much apart for the single data as for the pooled data, and the pooled model achieves better test results in all cases. The only exception to this rule is the medium PD dataset, where the single model achieves better and closer test and training results. However, if pooled runs outperform the single interventions in the training data, they consistently also outperform it in the test set.

#### Absolut evaluation

Regarding the absolute evaluation, all models achieve a balanced accuracy of more than 0.5 BACC, and are, therefore, better than chance. Further, 13 of the 27 models achieve a BACC on the test set of 0.67 or higher, with results differing across interventions and settings. For SAD, all but the single intervention small patient models achieve the threshold, with a maximum BACC of 0.74. For PD and MDD, not one model trained on the single intervention achieves clinically relevant prediction results. However, for MDD, both pooled model for the medium (0.67/0.67) and large (0.69/0.68) data achieve the threshold. For PD, only the largest balanced pooled model achieves clinically relevant prediction results on the test set.

## Discussion

Researching the heterogeneity in patient data for ICBTs for MDD, SAD, and PD, we found intervention-overarching patient groups in the first weeks of the interventions. Despite differing dropout rates per intervention (28–57%), the algorithm identified the respective most likely and least likely clusters to dropout. The active, middle and inactive clusters’ correspondence to low, middle and high dropout is in line with previous findings.^47^ Our first finding, that SAD, PD, and MDD patients have similar clusters of activity patterns may help the design and delivery of both individual and transdiagnostic interventions.

The answer to the first research question already hints toward the answer to the second; pooling the data was almost always favorable and doubled the likelihood of achieving clinically relevant test results. Most noticeably, having 873 mixed intervention training data points outperformed having 2304 individual intervention MDD or 1524 SAD patients. A possible hypothesis for this is that pooling different interventions forces the model to focus on general patterns rather than intervention-specific noise. Beyond better results, pooling data comes with the upside of less resources necessary for deploying and maintaining one versus three models. PD patients’ overall low results might partly be explained by their high class imbalance regarding dropouts and completers.

Two further interrelated key findings are the importance of independent test sets and risk of overfitting on small datasets. If the decision about whether to pool the data was made on the training CV scores, single intervention runs would have been preferred. Further, even with pooled data, in two out of three interventions the small datasets seemingly outperform the much larger datasets in the training score. This aligns with Sajjadian et al.'s^30^ findings that dataset size is significantly negatively correlated to the reported prediction accuracy. Our study's large test sets of 327–577 patients provides evidence that these good training results are biased as they fail to generalize. Sajjadian et al.^30^ further find that many studies do not even use an adequate training set up, instead relying on a single train-test split. As can be seen in the box plots, this can result in extremely high or low results, neither of which represent the expectable prediction performance. Making a deployment decision on such ungeneralizable training results comes with a myriad of problems: risk of suboptimal care, wasted resources and ultimately the corrosion of trust in the use of ML in clinical care.^7,30,63^ As this article shows, pooling different interventions enables providers to mitigate at least some of the risks when presented with the challenge of limited data availability.

The article, thus, contributes to e-mental health care by exploring the trade-off between data heterogeneity and dataset size and discussing the risk of overfitting.^30^

## Limitations

At the same time, several limitations apply. For one, the routine care data in this study only includes self-referred patients, which leaves it unclear if the insights generalize to different patient selection methods. Further, it is yet to be investigated if the similarities between patients translate to the same clinical actions against dropout being effective. Third, using k-means for the clustering analysis is an industry standard,^58^ but generative,^60^ or density-based methods^69^ may allow different insights. For the prediction task, the arguably biggest challenge in scaling the proposed approach is the availability of comparable interventions. While differing in content, the interventions at hand have a lot in common, the technical platform, the structure of treatments, the clinical routines for referral, assessment, therapist support, and the clinical staff. Therefore, our results do not warrant any prognosis about how the absence of these similarities would affect results. Lastly, this article neither compares the gains of pooled data to other options such as federate learning,^33^ nor offers definitive insights on what minimal dataset size is necessary to produce generalizable results. In the end, pooling data in the proposed way is only one possible tool in the attempt to produce more generalizable and useful prediction models in psychological research.

## Conclusion

Using ML to improve mental health care is a promising and growing research field. However, the lack of large datasets available hamper generalizability and cause biased results. This article addresses this issue by investigating the effects of pooling data from different interventions together to increase the training dataset size available.

A total of 6418 routine care patients’ data from ICBTs for depression, SAD, and PD is used to (1) investigate heterogeneity in patient online behavior between interventions and (2) analyze the benefits of data pooling when predicting intervention dropout. Regarding the first question, the cluster analysis suggests three intervention-overarching groups that are defined more by their online behavior and other clinical characteristics than by which ICBT-program they are in. The finding that patients across the three interventions have similar behavioral patterns is further supported in the prediction results. Ultimately, data pooling doubles the number of results that reach the threshold of clinical usefulness on the test set results. We, therefore, answer the second research question by concluding that data pooling is the superior approach based on our dataset.

## Supplemental Material

sj-docx-1-dhj-10.1177_20552076241248920 - Supplemental material for Dataset size versus homogeneity: A machine learning study on pooling intervention data in e-mental health dropout predictions Supplemental material, sj-docx-1-dhj-10.1177_20552076241248920 for Dataset size versus homogeneity: A machine learning study on pooling intervention data in e-mental health dropout predictions by Kirsten Zantvoort, Nils Hentati Isacsson, Burkhardt Funk and Viktor Kaldo in DIGITAL HEALTH

sj-docx-2-dhj-10.1177_20552076241248920 - Supplemental material for Dataset size versus homogeneity: A machine learning study on pooling intervention data in e-mental health dropout predictions Supplemental material, sj-docx-2-dhj-10.1177_20552076241248920 for Dataset size versus homogeneity: A machine learning study on pooling intervention data in e-mental health dropout predictions by Kirsten Zantvoort, Nils Hentati Isacsson, Burkhardt Funk and Viktor Kaldo in DIGITAL HEALTH

sj-xlsx-3-dhj-10.1177_20552076241248920 - Supplemental material for Dataset size versus homogeneity: A machine learning study on pooling intervention data in e-mental health dropout predictions Supplemental material, sj-xlsx-3-dhj-10.1177_20552076241248920 for Dataset size versus homogeneity: A machine learning study on pooling intervention data in e-mental health dropout predictions by Kirsten Zantvoort, Nils Hentati Isacsson, Burkhardt Funk and Viktor Kaldo in DIGITAL HEALTH

sj-xlsx-4-dhj-10.1177_20552076241248920 - Supplemental material for Dataset size versus homogeneity: A machine learning study on pooling intervention data in e-mental health dropout predictions Supplemental material, sj-xlsx-4-dhj-10.1177_20552076241248920 for Dataset size versus homogeneity: A machine learning study on pooling intervention data in e-mental health dropout predictions by Kirsten Zantvoort, Nils Hentati Isacsson, Burkhardt Funk and Viktor Kaldo in DIGITAL HEALTH
