# Supplementary material for: Dataset size versus homogeneity: A machine learning study on pooling intervention data in e-mental health dropout predictions
Source: Digit Health. 2024 May 15;10:20552076241248920. doi: 10.1177/20552076241248920 (PMC11097733; doi:10.1177/20552076241248920)
Supplement: sj-docx-1-dhj-10.1177_20552076241248920 - Supplemental material for Dataset size versus homogeneity: A machine learning study on pooling intervention data in e-mental health dropout predictions [file sj-docx-1-dhj-10.1177_20552076241248920.docx]

**Appendix 1 – Intervention Descriptions**

| **Module** | **Social Anxiety Disorder*** | **Major Depression Disorder** | **Panic Disorder** |
| --- | --- | --- | --- |
| 1 | Psychoeducation on cognitive behavioral therapy and social anxiety disorder. | Psychoeducation on cognitive behaviour therapy, and depression | Psychoeducation on cognitive behaviour therapy, anxiety, and panic disorder |
| 2 | Identifying negative thoughts and cognitive model of social anxiety disorder | Behavioural activation | Identifying negative automatic thoughts, the vicious circle of thoughts, emotions and internal focus |
| 3 | Behaviour experiments to challenge negative thoughts and reflect on treatment goals. | Behavioural activation (cont) | Cognitive restructuring |
| 4 | Exposure exercises and identifying safety behaviors | Cognitive restructuring | Interoceptive exposure |
| 5 | Exposure exercises (cont) identifying challenges | Cognitive restructuring (cont.) | Interoceptive exposure (continued) |
| 6 | Communication skills | Psychoeducation on and strategies for handling worry | Agoraphobic exposure |
| 7 | Exposure (cont) focus on social behaviours. | Psychoeducation on and strategies for handling insomnia | Agoraphobic exposure (continued) |
| 8 | Exposure (cont) | Behavioural activation and cognitive restructuring (cont.) | Agoraphobic exposure (continued) |
| 9 | Recapitulation of previous modules | Recapitulation of all previous modules, strategies for handling negative life events | Recapitulation of all previous  modules |
| 10 | Treatment summary and assistance of future planning. | Relapse prevention, long term goals | Relapse prevention |

* Before 2019, the SAD content was spread across 11 modules. However, the core content remained the same and were, in both variants, introduced in the first 8 modules. In both variants, the two or three later modules were focused on repetition and future maintenance only, because of which this is not assumed to have any impact on the study at hand. All patients were given 12 weeks to finish the intervention.
