# Supplementary material for: Dataset size versus homogeneity: A machine learning study on pooling intervention data in e-mental health dropout predictions
Source: Digit Health. 2024 May 15;10:20552076241248920. doi: 10.1177/20552076241248920 (PMC11097733; doi:10.1177/20552076241248920)
Supplement: sj-docx-2-dhj-10.1177_20552076241248920 - Supplemental material for Dataset size versus homogeneity: A machine learning study on pooling intervention data in e-mental health dropout predictions [file sj-docx-2-dhj-10.1177_20552076241248920.docx]

**Appendix 2 – Data pre-processing and description**

*Input variables.* The prediction is done after week four as a trade-off between gathering sufficient data versus maintaining sufficient time to intervene to prevent dropout. For the features, all data gathered after week 4 is disregarded to prevent target leak. To reduce cardinality of the interventions’ **start date**, only the year and a binary variable indicating the start in the fall/ winter vs. spring/ summer season are retrieved. Demographic variables consist of gender (three missing values) and age (five missing values) and for imputation, the majority class, and mean age are used. Each intervention targets a different disorder with a respective measurement for the primary symptoms. For PD, the Panic Disorder Severity Scale-Self Report (30,50,54), for SAD the Liebowitz Social Anxiety Scale-Self Report (37) and for MDD the Montgomery–Åsberg Depression Rating Scale-Self Report (38,39) are used. Within the time frame of the features, the symptom levels are logged at screening, right before starting the intervention in week 1 and at beginning of week 2, 3 and 4. Across all interventions, 286 (4%) screening, 135 (2%) week 1, 791 (12%) week 2, 612 (10%) week 3, and 698 (11%) week 4 data entries must be imputed. To retain the information of missing values, the count of missing symptom scores is recorded in an additional variable. Then, for any missing value, the next available symptom score of the respective patient is multiplied with the average change rate during that week. As all absolute symptom scores have a high autocorrelation between the times of measurement (0.73–0-84), only the pre intervention symptom value is kept as such. For the others, the relative change in symptoms since the last time of measurements is calculated, lowering correlation to -0.3-0.1. Less than 3.5% of the symptom values are 0, causing a zero-division problem during this transformation and are, therefore, replaced by a one for the calculation. As the ranges of the primary symptoms differ significantly from 0 to 28, 54 or 144, the pre symptom score is scaled based on the questionnaire maxima. In addition to the actual scores, the time needed to fill out the questionnaires is available if the questionnaire was filled out and missing values are imputed with the mean of the respective week. Further, patients were able to formulate free text in the exercises within the module, the message function to the therapist and the reflection homework as of module 2. For the exercise text data, double taps and non-characters were deleted before counting the number of characters. This step is mainly done to disregard the +/ - symbols the depression intervention generates for each entry in the positive or negative activity exercises. The character length of messages and the homework assignments are each summed for the first four weeks of the intervention.

To account for the therapist messages, the percentage of characters sent in the conversation produced by the therapist is included per week. In terms of the system-generated log in data, any session duration of less than 6 seconds is excluded to avoid recording accidental clicks or loading errors. Following related work, for the others, the sum of time spent on the intervention, the number of pages clicked, the number of sessions and number of days that patients logged in are recorded for week 1-4. In addition, the time patterns of the login behavior are gathered across all weeks by looking into the percentage of sessions per time of the week (Monday/ Tuesday, Wednesday/ Thursday, or Friday/ Saturday/ Sunday). Similarly, the sessions are counted for mornings (before 12 pm), daytime (12-6pm) and evenings (after 6pm). Essentially all patients start module 1 on day 0 but as patients work through the modules at slightly different paces the start date for the other modules differs. This information is kept by counting the days between the start of the intervention and the start of each module up to module 5. Any date after day 28 is replaced with 29 to indicate the start after the period of recording. Any patients that have single missing values for the patient behavior or message data (e.g., no recorded messages sent in the first week), are assumed to have a 0, as missing values indicate absence of activity. As common for online user behavior data, many of the variables have long tails, such that the highest five percent of values make up between 61% and nearly 100% of the variables range. To be able to better differentiate the large share of values in the lower end of the range, the highest 5% of values are replaced by the 95-Percentile of the training data plus the log of the respective value. This way, the order remains but most of the range is cut for all page (including symptom questionnaire) durations, number of pages, sessions, and character counts.

*Dropout definition.* Out of six adherence measures considered by Donkin, et al., (2011), module completion was the one included in most studies and with most consistently positive power towards explaining therapy outcome (9). Modules completed are inferred based on how many modules a patient has been given access to in combination with the number of homework assignments they handed in. For 22 patients, no reliable number could be computed due to missing values, because of which they were excluded from the analysis. Dropout has previously been defined as not having finished the minimum content deemed necessary for intended symptom improvement (9,47). However, as discussed by Beintner et al. (2019), the proposal of such minima is controversial and so far, lacks standards, resulting in a research question itself. Given the limitations of this paper, the minima are derived by looking into symptom improvements and the practical concern of class balance. In line with the definition of Beintner et al. (2019) the goal of the paper is to target those patients who are the most likely to be leaving the intervention too early to sufficiently benefit – *bad leavers*. At the same time, it is known that some patients require less time or content than assumed to improve their health and reach a satisfactory intervention outcome – *good leavers*. Allocating more resources to the latter patients would be detrimental to resource efficiency. Therefore, the interaction between incomplete interventions and health outcomes is investigated. The intervention success variable is based on a dichotomization of the primary symptom scale for each intervention. At the end of the intervention, the patient is labeled as an intervention success if either the score is below the absolute cut-off for remittance or if it is relatively 50% lower than the pre-treatment score (51). The remittance scores are 8 for PDSS-SR (48), 35 for LSAS-SR (49), and 11 for MADRS-S (50). For 502 patients, the outcome is unknown as they did not fill out any of the symptom questionnaires after week 8. The 50% improvement cutoff is included to account for the differences of improvements in dependence of the initial score as discussed by Karin et al (2018) (51). As these patients are of prime interest for dropout predictions, they are counted towards bad leavers. In the end, module eight is chosen as this cutoff contains all unknown leavers, includes all of the content introductions, as the last two modules are repetition and maintenance, and class balance is at a favorable 44% to 56% instead of 30% to 70% for module 7.

*Data description*. In the following, the remaining 1.631 panic disorder, 1.906 social anxiety, and 2.881 depression patients are described regarding their average values after pre-processing. The average age is 35.4 years (SD=11.38 years) and 63% are women. At 56%, slightly more than half of the patients start their intervention in the winter months and the mode year is 2014 at 710 patients.

PD patients have a mean pre-intervention symptom level of 11 (SD=5) on the 0-28 scale, MDD patients of 23 (SD=6) on the 0-54 scale and SAD of 72 (SD=24) on the 0-144 scale. This is a median change of -13% for PD, -10% for MDD, and +1% for SAD patients compared to their screening values. For the next weeks, symptoms have on average changed by another -17/ -6/ 0% for PD, -9/ -4/ -3% for MDD, and -3/ -2/ -2% for SAD patients.

In terms of messages, the average is 188/ 197 characters during the first two and third/ fourth weeks from the patients and 83-92% of the correspondence comes from therapists. Patients write on average 793 characters in the worksheet exercises in the first two weeks, and 706 and 563 respectively in the third and fourth week. The reflective homework exercises that are due after each module are on average 1.016/ 747/ 636/ 538 characters for weeks 1-4. Patients’ median modules completed during the first 28 days are 4, which corresponds to the weekly time plan. The median times between completing the modules 2/ 3/ 4 and the completion of the respective module before are 6/ 9/ 11 days. On average, patients spent 5.5/ 3.5/ 3.3/ 3.3 hours across 5/ 4/ 4/ 4 sessions that span over 4/ 3/ 3/ 3 days of the respective week. For 64% of individual patients 50% or more of the sessions fall in one of the week times categories, so either preferring the beginning of the week, the middle, or the weekend. Across all patients no preference is visible as each week category makes up 32-36% of sessions. For the times of the day, the hours during the day 12-18 o’clock (37%) and evenings past 18 o’clock (39%) are equally preferred over morning hours before noon (24%). Further, 82% of patients log in at least 50% of the time in one of the four daytimes. At 46%, almost half of patients are remitters, responders, or both. For 497 patients, the outcome is unknown as they do not have any of the later symptom questionnaires. The dropout rates differ across interventions, as 28% of panic disorder, 45% of depression and 57% of social anxiety patients are considered dropouts.
